# Supplementary material for: Exploring Seminal Plasma GSTM3 as a Quality and In Vivo Fertility Biomarker in Pigs—Relationship with Sperm Morphology
Source: Antioxidants (Basel). 2020 Aug 12;9(8):741. doi: 10.3390/antiox9080741 (PMC7466085; doi:10.3390/antiox9080741)
Supplement: Supplementary file 1 [file antioxidants-09-00741-s001.pdf]

Table S1: List of reagents, sources and identifiers used in the study.

Reagent	Source	Identifier
**5- and 6-chloromethyl-2 ,7-dichlorodihydrofluorescein diacetate acetyl ester (CM-H2DCFDA)**	Molecular Probes	C6827
**Anti-rabbit antibody conjugated with HRP**	Dako	P0448
**Bovine serum albumin (BSA)**	Roche Diagnostics	1,0735E+10
**Dimethyl sulfoxide (DMSO)**	Sigma-Aldrich	472301
**Fluorescein-conjugated peanut agglutinin (PNA-FITC)**	Sigma-Aldrich	L7381
**Formaldehyde**	Panreac	131328
**GSTM3 antibody**	Aviva Systems Biology	ARP53561_P050
**GSTM3 blocking peptide**	Aviva Systems Biology	AAP53561
**GSTM3 ELISA kit**	MyBioSource	MBS7260929
**Hoechst 33342 (H-42)**	Molecular Probes	H3570
**Immobilion™ Western Detection Reagents**	Millipore	WBULS0500
**Merocyanine 540 (M540)**	Molecular Probes	M24571
**Propidium iodide (PI)**	Molecular Probes	P3566
**Protease inhibitor cocktail**	Sigma-Aldrich	P8340
**Sample Buffer Laemmli 2×**	Sigma-Aldrich	S3401
**Sodium chloride (NaCl)**	Sigma-Aldrich	S9888
**Sodium orthovanadate (NO)**	Sigma-Aldrich	450243
**Tert-Butyl hydroperoxide solution**	Sigma-Aldrich	B2633
**Tris(hydroxymethyl)-aminomethane (Tris)**	Serva	37181.02
**TWEEN® 20**	Sigma-Aldrich	P1379
**xTractor™ Buffer kit**	Takara	635623
**YO-PRO™-1 Iodide (YO-PRO-1)**	Molecular Probes	Y3603

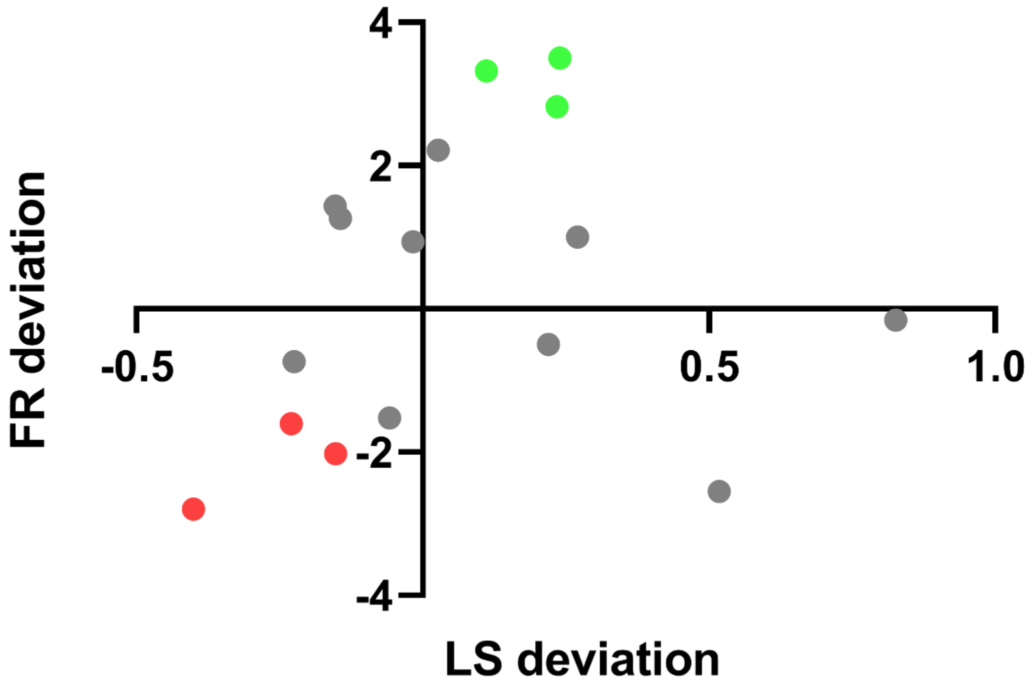

Figure S1 A dot plot of FR and LS deviation showing the selected individuals from the 16 AI-boars
